# Impact of the diagnosis of metabolic dysfunction-associated fatty liver disease and non-alcoholic fatty liver disease in patients undergoing liver transplantation for hepatocellular carcinoma

**DOI:** 10.3389/fendo.2024.1306091

**Published:** 2024-04-15

**Authors:** Ji-Qiao Zhu, Jia-Zong Liu, Shi-Wei Yang, Zhang-Yong Ren, Xiao-Yong Ye, Zhe Liu, Xian-Liang Li, Dong-Dong Han, Qiang He

**Affiliations:** ^1^ Department of Hepatobiliary and Pancreaticosplenic Surgery, Beijing Organ Transplant Center, Beijing Chaoyang Hospital, Capital Medical University, Beijing, China; ^2^ Department of Hepatobiliary Surgery, China-Japan Friendship Hospital, Beijing, China

**Keywords:** hepatocellular carcinoma, liver transplantation, non-alcoholic fatty liver disease, metabolic dysfunction-associated fatty liver disease, prognosis

## Abstract

**Purpose:**

Whether the diagnosis of non-alcoholic fatty liver disease or metabolic dysfunction-associated fatty disease has a different impact on liver transplant recipients with hepatocellular carcinoma is not yet clear.

**Methods:**

Data from a two-center retrospective cohort study were collected to compare and investigate the differences between non-alcoholic fatty liver disease and metabolic dysfunction-associated fatty liver disease in clinicopathologic parameters and prognosis among liver transplant recipients with hepatocellular carcinoma.

**Results:**

A total of 268 liver transplant recipients with hepatocellular carcinoma were included. The prevalence among pre- and post-transplant metabolic dysfunction-associated fatty liver disease was 10.82% and 30.22%, while for non-alcoholic fatty liver disease, it was 7.09% and 26.87%, respectively. The clinicopathological parameters were similar between the two pre-transplant groups. In contrast, the post-transplant group with metabolic dysfunction-associated fatty liver disease exhibited a higher prevalence of diabetes mellitus and a greater body mass index. However, the other parameters were similar between the two post-transplant groups (p > 0.05). Factors such as the largest tumor size > 4 cm, microvascular invasion, lack of tumor capsule, post-transplant metabolic dysfunction-associated fatty liver disease, and decreased post-transplant lymphocyte percentage were related to an increased risk of recurrence.

**Conclusion:**

In patients undergone liver transplantation for hepatocellular carcinoma, the diagnosis of metabolic dysfunction-associated fatty disease is more strongly associated with metabolic abnormalities than the diagnosis of non-alcoholic fatty liver disease and is an independent predictor of hepatocellular carcinoma recurrence.

## Introduction

Hepatocellular carcinoma (HCC), currently the sixth most common tumor worldwide, ranks fourth in terms of tumor mortality (1). Major risk factors contributing to the occurrence and development of HCC have been well established, including infection with hepatitis B virus (HBV) or hepatitis C virus (HCV), excessive alcohol consumption, and exposure to fungal metabolite (2, 3). Moreover, growing evidence suggests that metabolic risk factors, such as steatosis, obesity, and metabolic syndrome, can collectively contribute to the development of HCC, with a rising prevalence of HCC related to metabolic dysfunction (4, 5). Additionally, metabolic dysfunction can lead to concurrent diseases such as type 2 diabetes mellitus (T2DM), chronic kidney disease, cardiovascular disease (CVD), certain extrahepatic cancers, and severe liver-related complications (6). Non-alcoholic fatty liver disease (NAFLD), which is associated with metabolic dysfunction (7, 8), has emerged as a significant contributor to liver-related morbidity and mortality globally, with a prevalence of approximately 25% (9, 10). Moreover, NAFLD is one of the major indications for liver transplantation (10). However, NAFLD stands out in its diagnostic approach, often failing to account for the influence of coexisting metabolic dysfunction and various liver disease etiologies (11).

Recently, the concept of metabolic dysfunction-associated fatty liver disease (MAFLD) has been introduced by international consensus, focusing on its relevance to the underlying conditions of systemic metabolic dysfunction (12, 13). The new criteria define MAFLD as hepatic steatosis together with the presence of metabolic conditions (T2DM, obesity/overweight, or at least two metabolic abnormalities). Although MAFLD is presumed to have a stronger association with metabolic syndrome than NAFLD (14), understanding the application of this new terminology in liver transplantation remains limited. Moreover, the differential impacts of MAFLD and NAFLD on the pathological characteristics and outcomes of liver transplant recipients (LTR) with HCC have not been thoroughly explored in the existing literature.

Hence, we conducted a two-center retrospective study on LTR with HCC to investigate their clinicopathological data and prognosis, aiming to assess the respective influences of MAFLD and NAFLD on these patients.

## Materials and methods

### Study design and participants

This retrospective cohort study was conducted at Beijing Chaoyang Hospital (March 2011 and December 2021) and China-Japan Friendship Hospital (February 2018 and December 2021) involving LTR with HCC who underwent liver transplantation. The study was approved by the Institutional Review Board of both hospitals (No.2022-D-115) in accordance with the 1964 Helsinki Declaration and its later amendments. Informed consent was not required, given the retrospective nature of the study.

### Inclusion criteria and exclusion criteria

Inclusion criteria for LTR encompassed histopathologically confirmed HCC without distant metastases, undergoing liver transplantation, and a follow-up period of at least 6 months. Exclusion criteria consisted of combined-organ transplantation, liver retransplantation, presence of any other type of tumor, and missing data for a MAFLD diagnosis.

### Definitions

MAFLD diagnosis was based on hepatic steatosis in conjunction with one of the following three conditions: a body mass index (BMI) ≥ 23 kg/m^2^ in Asians, T2DM, or metabolic dysregulation (12, 13). T2DM was defined as a history of diabetes, and/or fasting plasma glucose ≥ 7.0 mmol/L, and/or two-hour post-load plasma glucose ≥ 11.1 mmol/L, and/or HbA1c ≥ 6.5% (15). Metabolic dysregulation was characterized by having at least two of the following metabolic conditions: waist circumference ≥ 90 in Asian men and ≥ 80 cm in Asian women; blood pressure ≥ 130/85 mmHg or receiving drug treatment; plasma triglycerides ≥ 1.70 mmol/L or receiving drug treatment; plasma high-density lipoprotein (HDL)-cholesterol < 1.0 mmol/L for males and < 1.3 mmol/L for females or receiving drug treatment; prediabetes (fasting plasma glucose levels 5.6-6.9 mmol/L, or two-hour post-load plasma glucose levels 7.8-11.0 mmol/L or HbA1c 5.7% to 6.4%).

NAFLD was diagnosed when hepatic steatosis was present, and secondary causes, such as excessive alcohol intake, viral hepatitis, autoimmune liver disease, parenteral nutrition, genetic disorders, hepatic malignancies, hepatobiliary infections, biliary tract diseases, medications, and starvation, were excluded (16). Alcohol intake was limited to ≤ 20 g/d for males and ≤ 10 g/d for females to define non-alcohol related liver conditions.

Hepatic steatosis was assessed through histopathology post-surgery for the recipient’s liver and via abdominal ultrasonography during follow-up for LTR. The diagnosis was conducted by two experienced pathologists and sonologists, who were blinded to each other’s evaluation.

### Data collection

Data collection was carried out through medical records and follow-up visits until December 2022. The following clinical variables were obtained: age, sex, alcohol intake, smoking (≥ 1 cigarette/d), body mass index, waist circumference, Child-Pugh grade, model of end-stage liver disease score (MELD score), immunosuppressive regimen, tumor-free survival, and overall survival in addition to histories of tumor therapy, CVD, hypertension and DM. CVD was comprised of coronary heart disease and stroke (17). Histopathological parameters included the largest tumor size, number of tumors, total tumor size, and tumor differentiation. The presence of tumor capsule, hepatic cirrhosis, hepatic capsule invasion, microvascular invasion, macrovascular invasion, and tumor within Milan criteria (18) was also noted. Laboratory measurements contained total bilirubin levels, aspartate aminotransferase levels, alanine transaminase levels, albumin levels, triglyceride levels, high-density lipoprotein cholesterol levels, fasting plasma glucose levels, alpha-fetoprotein levels, creatinine levels, neutrophil count, neutrophil percentage, lymphocyte count, lymphocyte percentage, neutrophil-to-lymphocyte count ratio.

### Follow-up

Patient follow-up visits were scheduled at three-month intervals during the first postoperative year, semi-annually during the second postoperative year, and annually thereafter. The follow-up period began on the day of patient discharge and ended either on the date of tumor recurrence or the closing date of follow-up. The primary objective of the study was to investigate the occurrence of MAFLD and NAFLD post-transplantation, with the secondary aim being to evaluate tumor-free survival.

### Statistical analysis

The normal distribution of continuous variables was tested using a Kolmogorov-Smirnov test. The independent samples t-test was employed for normally distributed variables, while the Wilcoxon ranksum test was selected for non-normally distributed variables. The Chi-square or Fisher’s exact test was used to compare categorical variables. The Kaplan-Meier method was used for survival analysis. The Cox regression model was employed for multifactor survival analysis. Data were analyzed using SPSS 19.0 computer software (IBM Corp., Armonk, NY, USA). The figures were generated using R 4.2.1 (https://www.R-project.org/). All statistical tests were two-sided, with statistical significance set at a P-value <0.05.

## Results

### Characteristics of LTR

A total of 268 patients with HCC, who underwent liver transplantation, were included in this study. The majority of patients were male (89.55%, n=234), with a mean age of 53.88 ± 9.05 years (range: 28~76 years). All LTR were of Asian descent. Prior to liver transplantation, 20.90% (n=56) of patients had DM while 17.91% (n=48) had hypertension. The mean BMI of LTR was 24.26 kg/m^2^. Following liver transplantation, the prevalence of DM and hypertension increased to 34.70% (n=93) and 27.61% (n=74), respectively, with a mean BMI of 23.42 kg/m^2^. During the follow-up period, 63 patients experienced tumor recurrence, while 204 LTR remained alive.

### Overlap between pre- and post-transplant MAFLD and NAFLD

**Figure 1:**
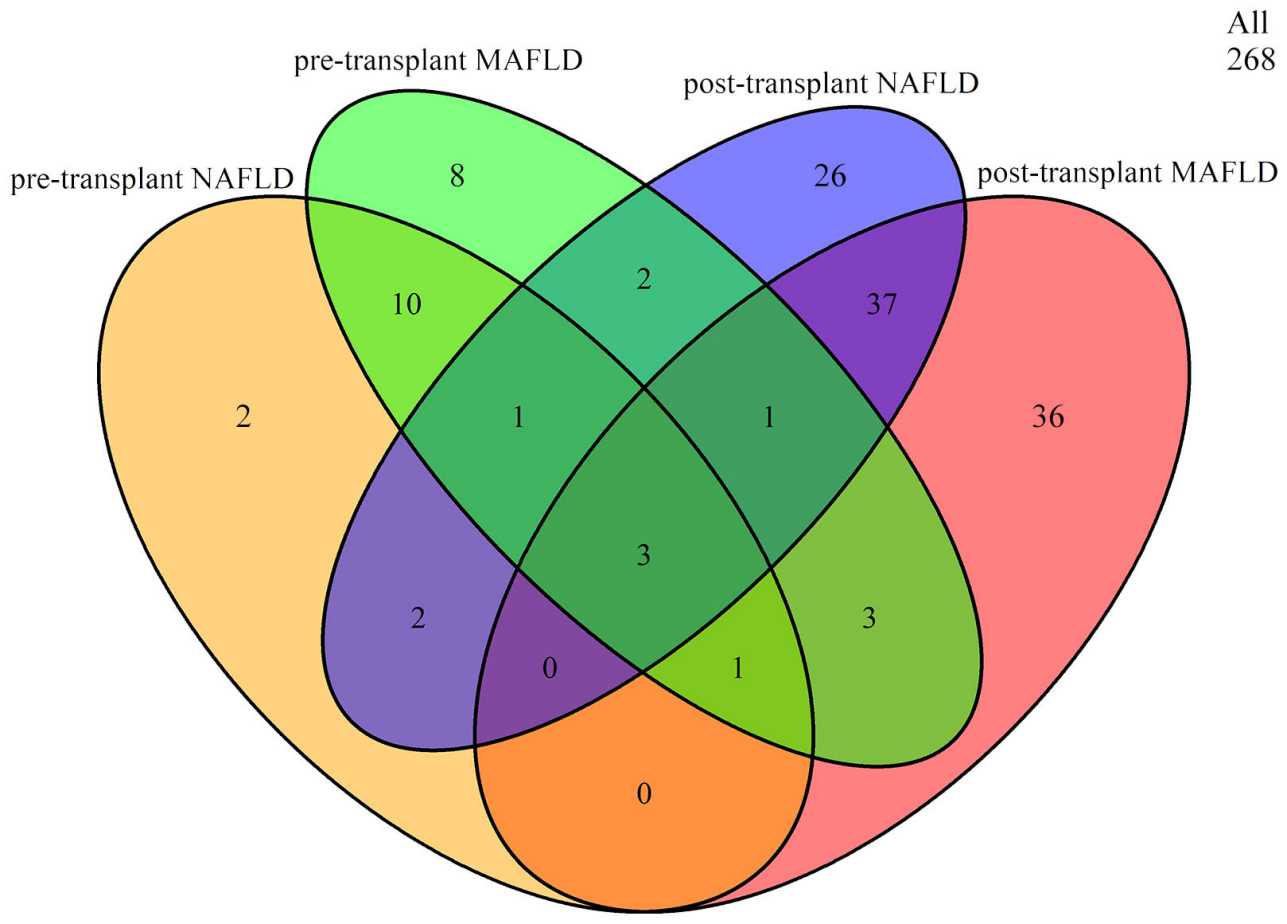
Overlap between pre- and post-transplant MAFLD and NAFLD. MAFLD, metabolic dysfunction-associated fatty liver disease; NAFLD, non-alcoholic fatty liver disease.

Before liver transplantation, 10.82% (29/268) of patients were diagnosed with MAFLD and 7.09% (19/268) with NAFLD. Among the 29 LTR with MAFLD, 15 also met the criteria for NAFLD. Following liver transplantation, the overall prevalence of MAFLD and NAFLD increased to 30.22% (81/268) and 26.87% (72/268), respectively. Among the 81 LTR with MAFLD, 41 also met the criteria for NAFLD. However, only a small number of LTR (n=3) fulfilled both criteria before and after liver transplantation (**Figure 1**).

### Comparison between pre-transplant MAFLD and NAFLD

The clinical characteristics of the pre-transplant MAFLD and NAFLD groups are described in **Table 1**. The two groups exhibited similar histopathological and laboratory features, including age, sex, Child-Pugh grade, MELD score, body mass index, waist circumference, history of tumor therapy, smoking, CVD, T2DM, hypertension, cirrhosis, largest tumor size, total tumor size, number of tumors, tumor differentiation, presence of tumor capsule, hepatic capsule invasion, microvascular invasion, macrovascular invasion, HCC within Milan criteria, serum albumin levels, serum creatinine levels, serum alpha-fetoprotein levels, triglyceride levels, HDL-cholesterol levels, fasting plasma glucose levels, neutrophil count, neutrophil percentage, lymphocyte count, lymphocyte percentage and neutrophil-to-lymphocyte count ratio (p > 0.05).

**Table 1:** Comparison of parameters between pre-transplant MAFLD and NAFLD.

Parameters	MAFLD (n=29)	NAFLD (n=19)	P value
Age (y)	56.03 ± 9.01	56.42 ± 7.97	0.880
Sex (male)	23	14	0.918
Child-Pugh grade			0.393
A	5	2	
B	13	6	
C	11	11	
MELD score	11.07 ± 7.59	12.79 ± 7.17	0.437
Body mass index (kg/m^2^)	24.01 ± 2.75	22.97 ± 2.57	0.195
Waist circumference (cm)	90.44 ± 6.48	88.31 ± 7.04	0.287
History of tumor therapy	9	5	0.725
Smoking	11	6	0.653
Cardiovascular disease	5	1	0.435
Type 2 diabetes mellitus	4	4	0.792
Hypertension	5	1	0.435
Cirrhosis	17	6	0.067
Largest tumor size ≤ 4 cm	15	6	0.169
Total tumor size ≤ 7 cm	19	13	0.835
Number of tumors ≤ 3	24	18	0.435
Differentiation			0.911
High	6	3	
Middle	20	14	
Low	3	2	
Tumor capsule	10	11	0.110
Hepatic capsule invasion	11	7	0.939
Microvascular invasion	4	0	0.091
Macrovascular invasion	1	1	1.000
Within Milan criteria	17	13	0.493
Albumin (g/L)	35.91 ± 6.63	36.27 ± 5.23	0.841
Creatinine (umol/L)	79.99 ± 37.47	79.62 ± 22.84	0.970
Alpha-fetoprotein (ng/mL)	118.79 ± 311.40	360.87 ± 984.14	0.899
Triglyceride (mmol/L)	2.00 ± 2.15	1.48 ± 0.83	0.328
HDL-cholesterol (mmol/L)	0.78 ± 0.41	0.84 ± 0.25	0.547
FPG (mmol/L)	6.74 ± 2.19	6.13 ± 2.05	0.339
Neutrophil count (10^9^/L)	3.29 ± 1.79	3.79 ± 2.13	0.389
Neutrophil percentage	65.92 ± 11.99	68.27 ± 11.34	0.502
Lymphocyte count (10^9^/L)	1.26 ± 0.75	1.36 ± 0.81	0.674
Lymphocyte percentage	25.96 ± 11.06	25.63 ± 9.65	0.916
NLR	3.57 ± 2.81	3.53 ± 2.55	0.958

MAFLD, metabolic dysfunction-associated fatty liver disease; NAFLD, non-alcoholic fatty liver disease; MELD, model for end-stage liver disease; HDL-cholesterol, high-density lipoprotein cholesterol; FPG, fasting plasma glucose; NLR, neutrophil-to-lymphocyte count ratio.

### Comparison between post-transplant MAFLD and NAFLD

**Figure 2:**
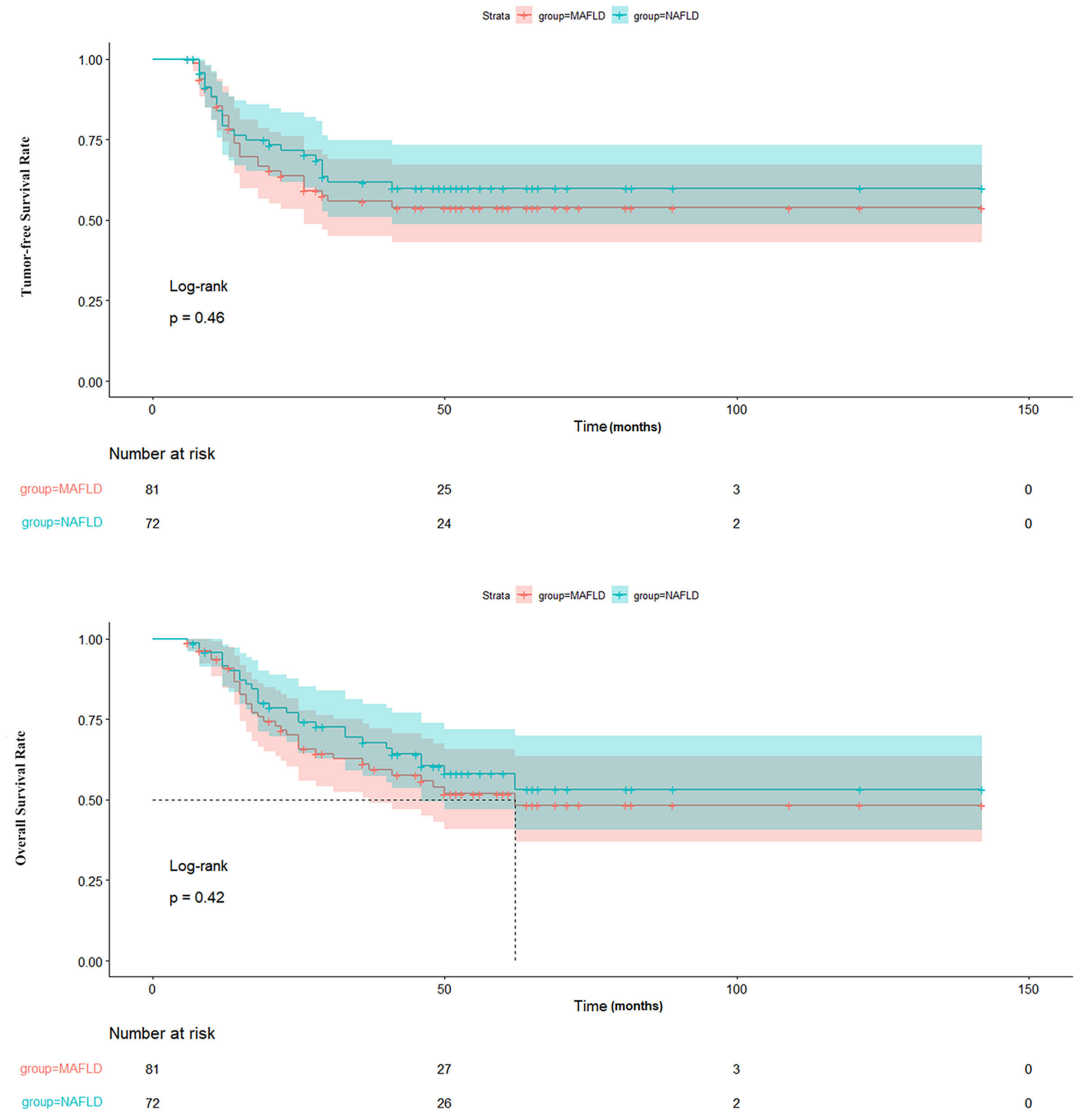
Comparison of TFS and OS between patients with MAFLD and NAFLD. TFS, tumor-free survival; OS, overall survival; MAFLD, metabolic dysfunction-associated fatty liver disease; NAFLD, non-alcoholic fatty liver disease.

A comparison of clinical parameters between the post-transplant MAFLD and NAFLD groups is presented in **Table 2**. The MAFLD group had a higher prevalence of DM (53% vs. 31%) and a greater BMI (25.53 ± 3.02 kg/m^2^ vs. 23.51 ± 3.53 kg/m^2^). Conversely, the NAFLD group displayed decreased waist circumference and fasting plasma glucose levels, although these differences did not reach statistical significance (p > 0.05). Other parameters, including age, sex, immunosuppressive regimen, smoking, CVD, hypertension, aspartate aminotransferase levels, alanine transaminase levels, total bilirubin levels, serum albumin levels, serum creatinine levels, alpha-fetoprotein levels, triglyceride levels, high-density lipoprotein cholesterol levels, neutrophil count, neutrophil percentage, lymphocyte count, lymphocyte percentage and neutrophil-to-lymphocyte count ratio were similar between the two groups (p > 0.05). In addition, the tumor-free survival and overall survival of the post-transplant MAFLD and NAFLD cohorts also showed no significant differences (p > 0.05) as illustrated in **Figure 2**.

**Table 2:** Comparison of parameters between post-transplant MAFLD and NAFLD.

Parameters	MAFLD (n=81)	NAFLD (n=72)	P value
Age (y)	55.65 ± 8.24	52.93 ± 9.73	0.063
Sex (male)	70	64	0.644
Immunosuppressive regimen			0.076
CNI	55	37	
mTOR inhibitor	3	7	
CNI+mTOR inhibitor	23	28	
Smoking	10	3	0.070
Cardiovascular disease	20	13	0.319
Type 2 diabetes mellitus	43	22	0.005
Hypertension	28	22	0.597
Body mass index (kg/m^2^)	25.53 ± 3.02	23.51 ± 3.53	0.000
Waist circumference (cm)	93.19 ± 8.68	90.45 ± 8.83	0.055
Aspartate aminotransferase (U/L)	37.72 ± 41.21	37.51 ± 39.14	0.523
Alanine transaminase (U/L)	31.94 ± 31.12	31.66 ± 29.83	0.921
Total bilirubin (umol/L)	22.93 ± 28.32	25.91 ± 30.54	0.223
Albumin (g/L)	40.47 ± 8.61	40.39 ± 9.21	0.828
Creatinine (umol/L)	88.65 ± 53.83	84.46 ± 38.41	0.814
Alpha-fetoprotein (ng/mL)	1100.16 ± 4386.87	607.59 ± 2832.93	0.398
Triglyceride (mmol/L)	1.97 ± 0.92	1.83 ± 0.84	0.304
HDL-cholesterol (mmol/L)	0.91 ± 0.34	0.97 ± 0.42	0.405
FPG (mmol/L)	6.77 ± 1.91	6.39 ± 1.96	0.054
Neutrophil count (10^9^/L)	3.97 ± 2.14	3.90 ± 2.09	0.828
Neutrophil percentage	63.25 ± 13.91	64.60 ± 14.91	0.564
Lymphocyte count (10^9^/L)	1.59 ± 1.18	1.50 ± 1.16	0.556
Lymphocyte percentage	25.92 ± 11.65	25.43 ± 11.69	0.796
NLR	3.32 ± 2.68	3.40 ± 2.26	0.390

MAFLD, metabolic dysfunction-associated fatty liver disease; NAFLD, non-alcoholic fatty liver disease; CNI, calcineurin inhibitor; mTOR inhibitor, mammalian target of rapamycin inhibitor; HDL-cholesterol, high-density lipoprotein cholesterol; FPG, fasting plasma glucose; NLR, neutrophil-to-lymphocyte count ratio.

### Risk factors associated with HCC recurrence

**Table 3** outlines the outcomes of the univariate analysis concerning risk factors linked with HCC recurrence. Individuals who experienced liver transplant rejection with recurrence were notably younger and demonstrated a higher incidence of tumors exceeding > 4 cm in size, total tumor size surpassing > 7 cm, and more than three tumors. Other risk factors included poor tumor differentiation, absence of tumor encapsulation, hepatic capsule invasion, microvascular invasion, macrovascular invasion, deviation from Milan criteria, post-transplant MAFLD and NAFLD, elevated levels of pre- and post-transplant alpha-fetoprotein, NLR, and decreased post-transplant lymphocyte percentage.

**Table 3:** Risk factors for HCC recurrence.

Parameters	Univariate analysis		
	With recurrence (N=63)	Without recurrence (N=205)	P value
Pre-surgery			
Age (y)	51.44 ± 9.25	54.62 ± 8.88	0.014
Sex (male)	55	185	0.504
Smoking	21	70	0.905
Drinking	19	53	0.500
History of tumor therapy	31	108	0.629
MELD score	9.17 ± 6.02	9.80 ± 6.87	0.227
Child-Pugh grade (A/B/C)	18/34/11	60/105/40	0.912
NAFLD	3	16	0.410
MAFLD	5	24	0.399
Cirrhosis	52	157	0.318
Largest tumor size ≤ 4 cm	19	112	0.001
Total tumor size ≤ 7 cm	20	143	0.000
Number of tumors ≤ 3	38	178	0.000
Differentiation (high/moderate/low)	5/39/19	45/128/32	0.005
Tumor capsule	11	63	0.039
Hepatic capsule invasion	29	46	0.000
Microvascular invasion	36	36	0.000
Macrovascular invasion	18	12	0.000
Within Milan criteria	18	127	0.000
Neutrophil count (10^9^/L)	3.72 ± 3.06	3.26 ± 2.28	0.192
Neutrophil percentage	67.07 ± 12.17	67.07 ± 13.28	0.997
Lymphocyte count (10^9^/L)	1.07 ± 0.64	1.01 ± 0.68	0.537
Lymphocyte percentage	23.11 ± 11.15	22.95 ± 11.41	0.919
NLR	6.41 ± 11.50	5.56 ± 10.49	0.586
Alpha-fetoprotein (ng/mL)	3057.89 ± 6464.33	470.74 ± 1957.62	0.000
Post-surgery			
Smoking	2	19	0.115
Drinking	18	40	0.127
Hepatitis recurrence	11	25	0.284
Immunosuppressive regimen (CNI/mTOR/both)	41/7/15	107/20/78	0.113
NAFLD	26	46	0.003
MAFLD	32	49	0.000
Neutrophil count (10^9^/L)	3.77 ± 1.60	3.83 ± 1.94	0.817
Neutrophil percentage	65.73 ± 13.29	63.22 ± 12.45	0.169
Lymphocyte count (10^9^/L)	1.37 ± 1.02	1.57 ± 0.83	0.124
Lymphocyte percentage	23.03 ± 11.43	27.18 ± 10.10	0.006
NLR	3.92 ± 3.04	3.05 ± 2.96	0.048
Alpha-fetoprotein (ng/mL)	2417.74 ± 5994.92	56.32 ± 426.89	0.003

HCC, hepatocellular carcinoma; MELD, model for end-stage liver disease; NAFLD, non-alcoholic fatty liver disease; MAFLD, metabolic dysfunction-associated fatty liver disease; NLR, neutrophil-to-lymphocyte count ratio; CNI, calcineurin inhibitor; mTOR inhibitor, mammalian target of rapamycin inhibitor.

**Figure 3:**
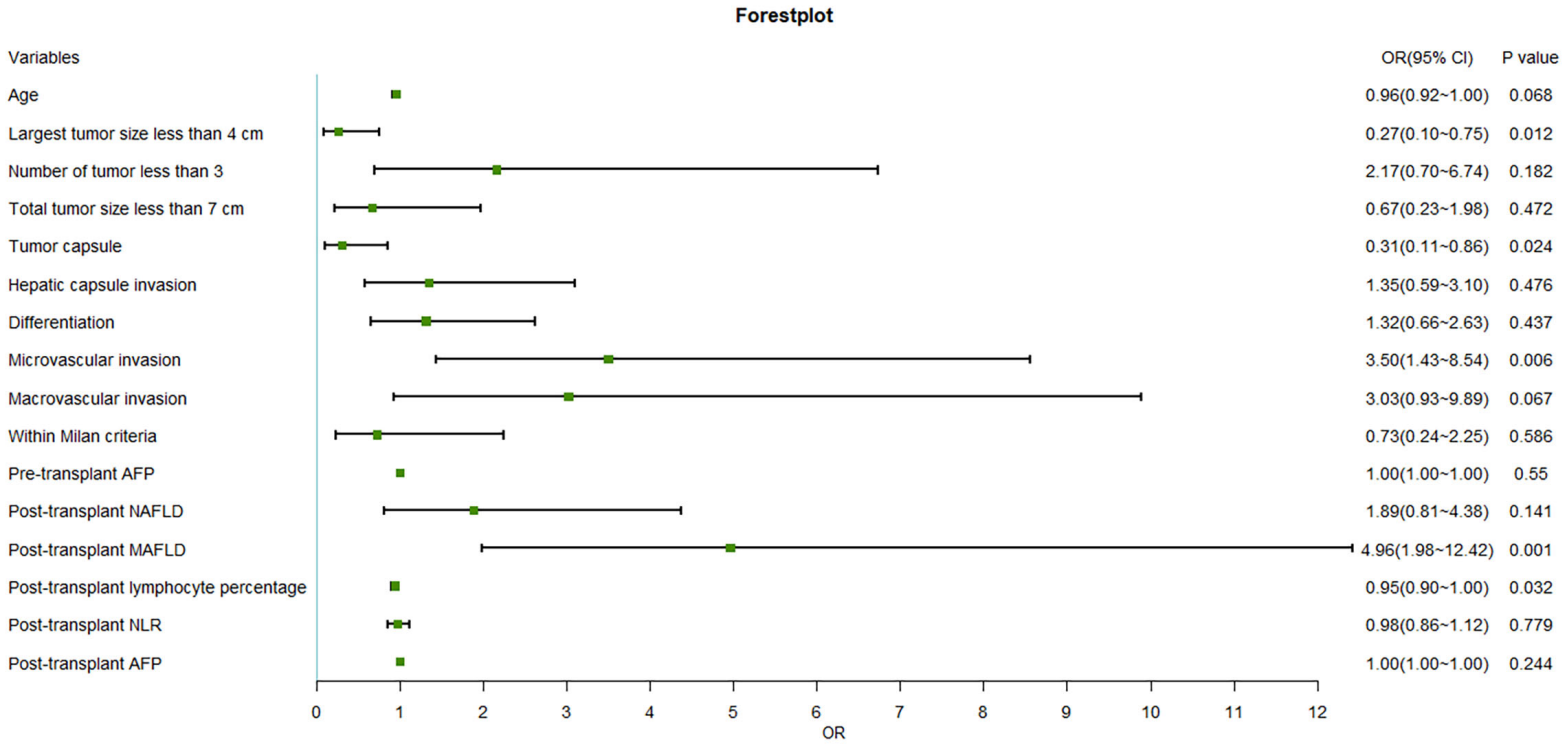
Multivariate analysis of prognostic factors related to HCC recurrence. HCC, hepatocellular carcinoma; AFP, alpha-fetoprotein; NAFLD, non-alcoholic fatty liver disease; MAFLD, metabolic dysfunction-associated fatty liver disease; NLR, neutrophil-to-lymphocyte count ratio.

The stepwise Cox proportional hazard model depicted in **Figure 3** summarizes the prognostic factors associated with HCC recurrence in this cohort. The largest tumor size (OR = 0.27, p = 0.012), microvascular invasion (OR = 3.50, p = 0.006), absence of tumor capsule (OR = 0.31, p = 0.024), post-transplant MAFLD (OR = 4.96, p = 0.001), and decreased post-transplant lymphocyte percentage (OR = 0.95, p = 0.032) were identified as factors associated with a higher risk of recurrence.

**Figure 4:**
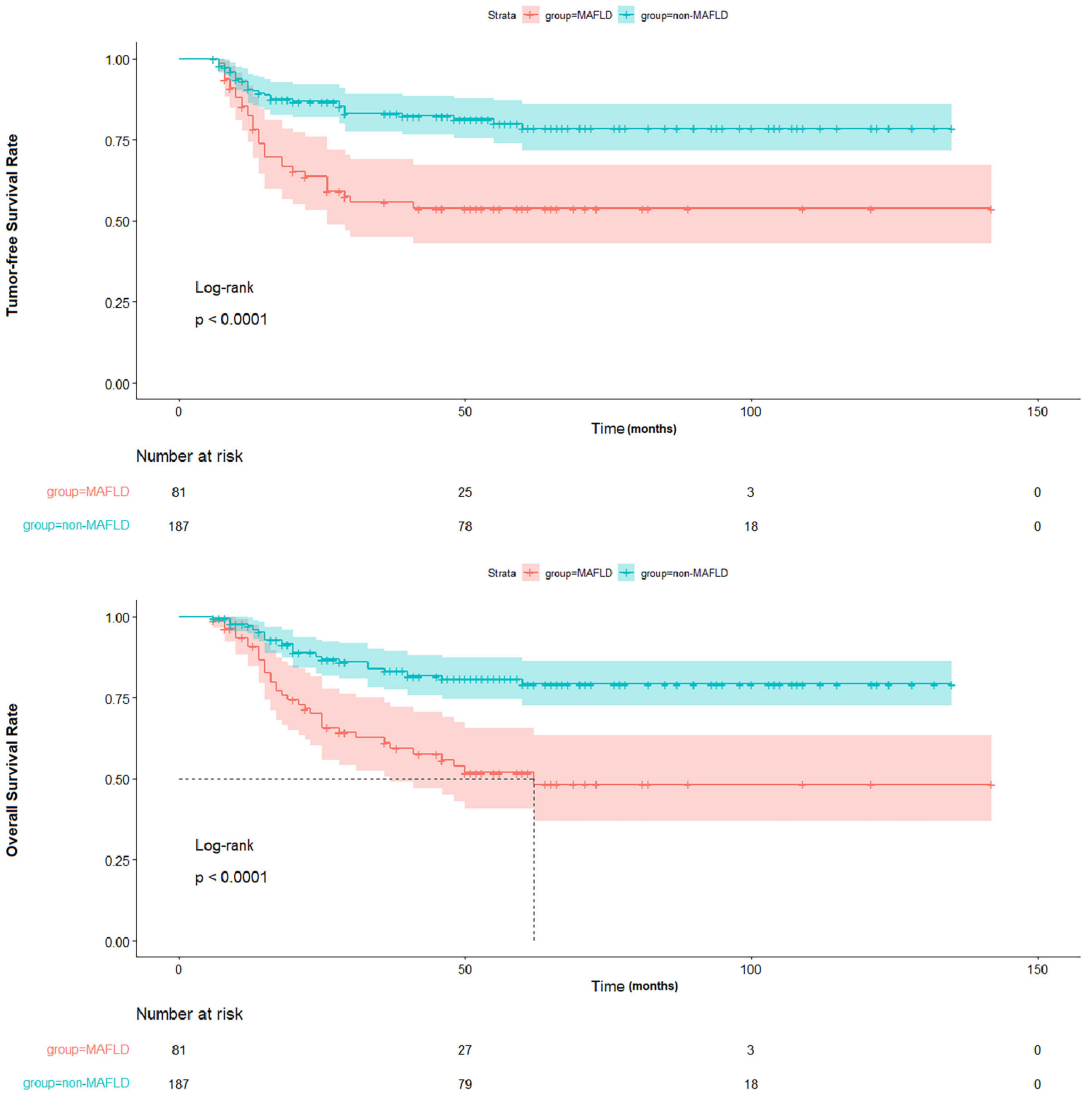
Comparison of TFS and OS between patients with and without MAFLD. TFS, tumor-free survival; OS, overall survival; MAFLD, metabolic dysfunction-associated fatty liver disease.

To further ascertain the impact of post-transplant MAFLD on tumor recurrence, the tumor-free survival of liver transplant recipients with and without recurrence was evaluated. The findings revealed that post-transplant MAFLD was indicative of patients at a heightened risk of recurrence, resulting in significantly decreased tumor-free survival rates (p < 0.001) and overall survival rates (p < 0.001) as depicted in **Figure 4**.

## Discussion

We conducted a comparative analysis between LTR with MAFLD and NAFLD in terms of prevalence and characteristics. Our findings indicate that LTR with post-transplant MAFLD are significantly associated with metabolic risk factors such as T2DM and obesity. Additionally, we identified several independent risk factors for HCC recurrence in LTR with HCC, including post-transplant MAFLD, the largest tumor size > 4 cm, microvascular invasion, absence of tumor capsule, and decreased post-transplant lymphocyte percentage.

The term MAFLD was introduced as a replacement for NAFLD to more accurately reflect the pathology of liver disease, recognizing the limitations of the NAFLD definition (12, 13). Since then, substantial efforts have been devoted to exploring the relationship between NAFLD and MAFLD. Lim et al. found significant differences in the natural progression of MAFLD and NAFLD (19), while Lin et al. revealed that patients with MAFLD had a higher risk of disease progression (14). However, it remains unknown whether NAFLD and MAFLD can be used interchangeably to characterize LTR with HCC. This study revealed that the prevalence of MAFLD was higher than that of NAFLD before and after transplantation. Surprisingly, only a small proportion of patients with pre-transplant MAFLD (10.82%) and NAFLD (7.09%) received a diagnosis, resulting in an overlap of 52% among LTR with MAFLD. However, a meta-analysis of 379,801 patients reported a pooled prevalence of 39.22% for MAFLD and 33.86% for NAFLD, with regional variations. Interestingly, among patients with MAFLD, the pooled prevalence of those with both MAFLD and NAFLD was remarkably high at 81.59% (19). However, LTR constituted a distinct patient population with unique pathophysiologic characteristics. It is evident that the majority of LTR with HCC in this study were caused by hepatitis infection and excessive alcohol intake. Consequently, upon admission, certain patients exhibited severe cirrhosis and liver function decompensation, as determined by high MELD scores and Child-Pugh grade B or C ratios. These factors might result in malnutrition and a decrease in lymphocyte subset percentages (20), thereby significantly reducing the number of both MAFLD and NAFLD cases. Therefore, the characteristics of pre-transplant MAFLD and NAFLD in LTR with HCC were found to be similar upon comparison, suggesting the interchangeability of these two terms in a clinical setting.

Following transplantation, the prevalence of MAFLD and NAFLD increased due to the amelioration of malnutrition with the introduction of a healthier liver. Furthermore, urbanization and the use of immunosuppressant drugs can also contribute to the elevated prevalence (9, 21–23). while the routine practice at our center for LTR with HCC involved early corticosteroid withdrawal, high doses of corticosteroids were administered along with other immunosuppressant drugs in cases of acute rejection, causing insulin resistance and weight gain (24, 25). Nevertheless, concurrent recurrence of hepatitis and alcohol consumption may lead to an increase in MAFLD cases while reducing the number of NAFLD cases based on their respective definitions. Hepatitis C recurrence in some LTR can increase the risk of dyslipidemia, hepatic steatosis, and insulin resistance, whereas alcohol intake is strongly associated with hepatic steatosis and dyslipidemia, although further research is needed to investigate the link between HBV and metabolic syndrome (26–28). Moreover, compared to the NAFLD group, LTR with MAFLD were more strongly associated with metabolic disorders, including a higher proportion of individuals with T2DM and a higher BMI, which are key criteria for diagnosing MAFLD. Other characteristics did not exhibit significant differences, as MAFLD and NAFLD share a similar pathophysiology involving the metabolic functions of the liver, particularly an extended endoplasmic reticulum network (29). Consequently, there was an overlap of 41 patients (51%) among LTR with MAFLD, which is still lower than the prevalence (81.59%) reported in a meta-analysis (19).

Finally, we identified five independent prognostic factors for HCC recurrence in this study. Metabolic syndrome has been found to be a significant risk factor for HCC (4, 5). Since the novel definition of MAFLD was proposed based on metabolic syndrome, post-transplant MAFLD can effectively stratify LTR at a high risk of HCC recurrence. Thus MAFLD was revealed as an independent risk factor for HCC recurrence in our study. Additionally, our study further confirmed the significant association between the largest tumor size and microvascular invasion, traditionally indicators of aggressive biology (30, 31), with HCC recurrence. Furthermore, the presence of the tumor capsule exhibited a protective effect by acting as a barrier against vascular and local invasion (32, 33). Lastly, LTR with a low post-transplant lymphocyte percentage showed a worse prognosis, as lymphocytes play a critical role in tumor surveillance (34, 35). Consequently, decreased post-transplant lymphocyte percentages weakened the antitumor response in these patients, leading to HCC recurrence.

There are several limitations in this study. Firstly, the sample size of patients with pre- and post-transplant MAFLD and NAFLD is small, considering the substantial overlap between the two conditions. This study involved a unique population with a low prevalence of pre-transplant MAFLD and NAFLD, strongly associated with the presence of cirrhosis. Regarding post-transplant MAFLD and NAFLD, there are several conceivable explanations. LTR paid more attention to their health post-transplantation, adhering to regular follow-ups and timely treatment of emerging diseases. Besides, patients with missing data required for MAFLD classification were excluded from the study, which further decreased the prevalence of MAFLD. Hence, a two-center cohort study was conducted to increase patient enrollment. Moreover, with an increase in the number of LTR experiencing post-transplant MAFLD or NAFLD, the occurrence of LTR with overlapping diseases also escalated. Secondly, ultrasonography-based assessment was utilized instead of biopsy-based assessment for diagnosing post-transplant MAFLD and NAFLD because the latter is invasive and carries the risk of severe complications. However, the diagnosis based on ultrasound features may have certain limitations due to its dependency on operator skills. Consequently, two experienced sonologists, blinded to each other’s evaluations, performed the diagnosis to minimize potential bias. Thirdly, a significant number of LTR with HCC exceeding Milan criteria had a history of tumor therapy, potentially impacting hepatic steatosis. Additionally, the MAFLD definition was based on Asian criteria. Therefore, the results of this study should be interpreted with caution for Caucasian men/women. The retrospective design of this study calls for future randomized controlled trials in multiple centers to validate these findings and assess reproducibility in Caucasian populations.

In summary, patients with MAFLD have a stronger association with fatty liver disease compared to those with NAFLD following liver transplantation. Post-transplant MAFLD has the potential to stratify patients based on tumor progression.

## Data availability statement

Data analyzed in the study are included in the article/supplementary material. Further inquiries can be directed to the corresponding authors.

## Ethics statement

The studies involving humans were approved by the Institutional Review Board of Beijing Chaoyang Hospital and the Institutional Review Board of China-Japan Friendship Hospital. The studies were conducted in accordance with the local legislation and institutional requirements. The ethics committee/institutional review board waived the requirement of written informed consent for participation from the participants or the participants’ legal guardians/next of kin because this was a two-center retrospective cohort study.

## Author contributions

J-QZ: Data curation, Writing – original draft. J-ZL: Data curation, Writing – original draft. S-WY: Data curation, Writing – original draft. Z-YR: Formal analysis, Writing – review & editing. X-YY: Formal analysis, Writing – review & editing. ZL: Investigation, Writing – review & editing. X-LL: Investigation, Writing – review & editing. D-DH: Conceptualization, Writing – review & editing. QH: Conceptualization, Writing – review & editing.
